# Supplementary material for: ProteinShader: illustrative rendering of macromolecules
Source: BMC Struct Biol. 2009 Mar 30;9:19. doi: 10.1186/1472-6807-9-19 (PMC2672931; doi:10.1186/1472-6807-9-19)
Supplement: Additional file 1 — ProteinShader program without source code. This compressed file contains the complete ProteinShader program including associated libraries, but no source code. A README.txt file gives an overview of the ProteinShader distribution, and the index.html file in the help subdirectory has directions on getting started with the program as well as a set of tutorials. [file 1472-6807-9-19-S1.zip › ProteinShader-beta-0_9_4-binary/help/api/org/proteinshader/structure/io/enums/CategoryEnum.html]

CategoryEnum (ProteinShader API)


|  |  |  |
| --- | --- | --- |
| **Overview** \| **Package** \| **Class** \| **Use** \| **Tree** \| **Deprecated** \| **Index** \| **Help** | |  |
| PREV CLASS   **NEXT CLASS** | **FRAMES**    **NO FRAMES**     **All Classes** |  |
| SUMMARY: NESTED \| ENUM CONSTANTS \| FIELD \| METHOD | DETAIL: ENUM CONSTANTS \| FIELD \| METHOD |  |


---


## org.proteinshader.structure.io.enums Enum CategoryEnum

```
java.lang.Object
  java.lang.Enum<CategoryEnum>
      org.proteinshader.structure.io.enums.CategoryEnum
```

**All Implemented Interfaces:**: Serializable, Comparable<CategoryEnum>

---

``` public enum CategoryEnum extends Enum<CategoryEnum> ```

This file provides an enumeration of the categories that RecordEnums
fit into. The categories are based on sections in the 
Protein Data Bank Content Guide (Version 2.2, 20 Dec 1996).

---

**Enum Constant Summary**

`BOOKKEEPING`
  
          The known RecordEnums in this category are MASTER and END.

`CONNECTIVITY`
  
          The known RecordEnum in this category is CONECT.

`CONNECTIVITY_NOTES`
  
          The known RecordEnums in this category are SSBOND, LINK, HYDBND,
SLTBRG, and CISPEP.

`COORDINATES`
  
          The known RecordEnums in this category are MODEL, ATOM, SIGATM,
ANISOU, SIGUIJ, TER, HETATM, and ENDMDL.

`HETEROGEN_INFO`
  
          The known RecordEnums in this category are HET, HETNAM, HETSYN,
and FORMUL.

`MISCELLANEOUS`
  
          The known RecordEnum in this category is SITE (FTNOTE, which is no
longer used, has been placed in this category only for lack of a
better place to add it).

`SECONDARY_STRUCTURE`
  
          The known RecordEnums in this category are HELIX, SHEET, and TURN.

`SEQUENCE_INFO`
  
          The known RecordEnums in this category are DBREF, SEQADV, SEQRES,
and MODRES.

`TITLE_SECTION`
  
          The known RecordEnums in this category are HEADER, OBSLTE, TITLE,
CAVEAT, COMPND, SOURCE, KEYWORDS, EXPDTA, AUTHOR, REVDAT, SPRSDE,
JRNL, and REMARK.

`TRANSFORMATIONS`
  
          The known RecordEnums in this category are CRYST1, ORIGX1, ORIGX2,
ORIGX2, SCALE1, SCALE2, SCALE3, MTRIX1, MTRIX2, MTRIX3, and TVECT.


| **Method Summary** | |
| --- | --- |
| `String` | `getName()`             Returns the name of the category as a String suitable for use in a menu. |
| `static CategoryEnum` | `valueOf(String name)`             Returns the enum constant of this type with the specified name. |
| `static CategoryEnum[]` | `values()`             Returns an array containing the constants of this enum type, in the order they're declared. |

**Methods inherited from class java.lang.Enum**

`clone, compareTo, equals, getDeclaringClass, hashCode, name, ordinal, toString, valueOf`

**Methods inherited from class java.lang.Object**

`finalize, getClass, notify, notifyAll, wait, wait, wait`

**Enum Constant Detail**

### TITLE\_SECTION

```
public static final CategoryEnum TITLE_SECTION
```

:   The known RecordEnums in this category are HEADER, OBSLTE, TITLE,
    CAVEAT, COMPND, SOURCE, KEYWORDS, EXPDTA, AUTHOR, REVDAT, SPRSDE,
    JRNL, and REMARK.

---


### SEQUENCE\_INFO

```
public static final CategoryEnum SEQUENCE_INFO
```

:   The known RecordEnums in this category are DBREF, SEQADV, SEQRES,
    and MODRES.

---


### HETEROGEN\_INFO

```
public static final CategoryEnum HETEROGEN_INFO
```

:   The known RecordEnums in this category are HET, HETNAM, HETSYN,
    and FORMUL.

---


### SECONDARY\_STRUCTURE

```
public static final CategoryEnum SECONDARY_STRUCTURE
```

:   The known RecordEnums in this category are HELIX, SHEET, and TURN.

---


### CONNECTIVITY\_NOTES

```
public static final CategoryEnum CONNECTIVITY_NOTES
```

:   The known RecordEnums in this category are SSBOND, LINK, HYDBND,
    SLTBRG, and CISPEP.

---


### MISCELLANEOUS

```
public static final CategoryEnum MISCELLANEOUS
```

:   The known RecordEnum in this category is SITE (FTNOTE, which is no
    longer used, has been placed in this category only for lack of a
    better place to add it).

---


### TRANSFORMATIONS

```
public static final CategoryEnum TRANSFORMATIONS
```

:   The known RecordEnums in this category are CRYST1, ORIGX1, ORIGX2,
    ORIGX2, SCALE1, SCALE2, SCALE3, MTRIX1, MTRIX2, MTRIX3, and TVECT.

---


### COORDINATES

```
public static final CategoryEnum COORDINATES
```

:   The known RecordEnums in this category are MODEL, ATOM, SIGATM,
    ANISOU, SIGUIJ, TER, HETATM, and ENDMDL.

---


### CONNECTIVITY

```
public static final CategoryEnum CONNECTIVITY
```

:   The known RecordEnum in this category is CONECT.

---


### BOOKKEEPING

```
public static final CategoryEnum BOOKKEEPING
```

:   The known RecordEnums in this category are MASTER and END.


**Method Detail**

### values

```
public static final CategoryEnum[] values()
```

:   Returns an array containing the constants of this enum type, in
    the order they're declared. This method may be used to iterate
    over the constants as follows:

    ```
    for(CategoryEnum c : CategoryEnum.values())
            System.out.println(c);
    ```

    :   **Returns:**: an array containing the constants of this enum type, in the order they're declared

---


### valueOf

```
public static CategoryEnum valueOf(String name)
```

:   Returns the enum constant of this type with the specified name.
    The string must match *exactly* an identifier used to declare an
    enum constant in this type. (Extraneous whitespace characters are
    not permitted.)

    :   **Parameters:**: `name` - the name of the enum constant to be returned. **Returns:**: the enum constant with the specified name **Throws:**: `IllegalArgumentException` - if this enum type has no constant with the specified name

---


### getName

```
public String getName()
```

:   Returns the name of the category as a String suitable for use in a
    menu.

    :   **Returns:**: The category name in lowercase letters, but with the first letter of each word capitalized.


---


|  |  |  |
| --- | --- | --- |
| **Overview** \| **Package** \| **Class** \| **Use** \| **Tree** \| **Deprecated** \| **Index** \| **Help** | |  |
| PREV CLASS   **NEXT CLASS** | **FRAMES**    **NO FRAMES**     **All Classes** |  |
| SUMMARY: NESTED \| ENUM CONSTANTS \| FIELD \| METHOD | DETAIL: ENUM CONSTANTS \| FIELD \| METHOD |  |


---

# *Copyright © 2007-2008*
